# GSK-3β phosphorylation of functionally distinct tau isoforms has differential, but mild effects

**DOI:** 10.1186/1750-1326-4-18

**Published:** 2009-05-02

**Authors:** Kellen Voss, T Chris Gamblin

**Affiliations:** 1 Department of Molecular Biosciences, University of Kansas, 1200 Sunnyside Avenue, Lawrence, KS 66045, USA

## Abstract

**Background:**

Tau protein exists as six different isoforms that differ by the inclusion or exclusion of exons 2, 3 and 10. Exon 10 encodes a microtubule binding repeat, thereby resulting in three isoforms with three microtubule binding repeats (3R) and three isoforms that have four microtubule binding repeats (4R). In normal adult brain, the relative amounts of 3R tau and 4R tau are approximately equal. These relative protein levels are preserved in Alzheimer's disease, although in other neurodegenerative tauopathies such as progressive supranuclear palsy, corticobasal degeneration and Pick's disease, the ratio of 3R:4R is frequently altered. Because tau isoforms are not equally involved in these diseases, it is possible that they either have inherently unique characteristics owing to their primary structures or that post-translational modification, such as phosphorylation, differentially affects their properties.

**Results:**

We have determined the effects of phosphorylation by a kinase widely believed to be involved in neurodegenerative processes, glycogen synthase kinase-3β (GSK-3β), on the microtubule binding and inducer-initiated polymerization of these isoforms in vitro. We have found that each isoform has a unique microtubule binding and polymerization profile that is altered by GSK-3β. GSK-3β phosphorylation had differential effects on the isoforms although there were similarities between isoforms and the effects were generally mild.

**Conclusion:**

These results indicate that tau phosphorylation by a single kinase can have isoform specific outcomes. The mild nature of these changes, however, makes it unlikely that differential effects of GSK-3β phosphorylation on the isoforms are causative in neurodegenerative disease. Instead, the inherent differences in the isoform interactions themselves and local conditions in the diseased cells are likely the major determinant of isoform involvement in various neurodegenerative disorders.

## Background

Tauopathies, such as Alzheimer's disease (AD), are characterized by the abnormal aggregation of a hyperphosphorylated microtubule associated protein, tau. The aggregation and phosphorylation of tau correlates with neurodegenerative changes in these diseases (reviewed in [1]). It is therefore generally believed that aggregation and phosphorylation changes are causative factors in the etiology of tauopathies. One complicating factor to these observations is that tau mRNA is alternatively spiced to give rise to six protein isoforms. The isoforms are generated by splicing of exons 2, 3 and 10, which encodes for microtubule binding repeat 2, giving rise to isoforms with three microtubule binding repeats (3R) or four (4R) [2]. The levels of 3R and 4R tau do not differ between normal and AD brains, but in both cases the levels of alternatively spliced N-terminal exons differ. Isoforms with only exon 2 are the most abundant, followed by isoforms without N-terminal exons and isoforms with both exons 2 and 3 are the least abundant [3]. In other tauopathies, the ratio of 3R isoforms to 4R isoforms can be altered (reviewed in [4]). For example, progressive supranuclear palsy (PSP) can be accompanied by increases in 4R tau, while Pick's Disease is often characterized by an increased amount of 3R isoforms (reviewed in [4]). These increases have been explained in part by intronic mutations that promote or hinder splicing of exon 10, but have no effect on the protein sequence (reviewed in [5]). The above observations suggest that a shift in isoform expression may contribute to neurodegenerative changes in tauopathies. However, the individual contributions of tau isoforms to the neurodegenerative process are not well understood. One possibility is that the inherent differences between isoforms are sufficient to cause neurodegenerative changes when their relative levels are altered.

Tau polymerization into filaments similar to those observed in neurodegenerative diseases can be modeled in vitro by the addition of inducer molecules such as arachidonic acid (ARA) and heparin (reviewed in [6]) in a complicated fashion [7]. It is currently unknown whether different isoforms share ARA and heparin-induced polymerization profiles similar to 2N4R tau.

In addition to being abnormally polymerized, tau has been shown to be hyperphosphorylated in AD pathology. "Hyperphosphorylated" is a loosely defined term used to describe abnormally or inappropriately phosphorylated tau in AD. Although there is not a strict definition, some properties of hyperphosphorylated tau include altered electrophoretic mobility, conformational changes, increased levels of phosphate incorporation, and site-specific inappropriate phosphorylation [8-10]. While it is not known how hyperphosphorylated tau is generated, glycogen synthase kinase-3β (GSK-3β) is widely believed to play a role in this process (reviewed in [10]). In vitro, GSK-3β phosphorylates tau at 14 sites that overlap with AD-tau, as determined by mass spectrometry, and phosphorylation site-specific antibodies [11-14]. In vivo, treatment of transgenic mouse models of tau-induced neurodegeneration with lithium chloride, an inhibitor of GSK-3β, reduces both tau phosphorylation and resulting tau pathology [15,16]. Phosphorylation of 2N4R tau in vivo by GSK-3β has reduced affinity for microtubules [17] and phosphorylation of 0N3R tau in vitro by GSK-3β shows mild changes in affinity of tau for microtubules [18]. Recombinant tau phosphorylated by GSK-3β, upon polymerization with an inducer, forms structures that resemble neurofibrillary tangles in vitro [13,19]. However, it is not known how GSK-3β phosphorylation affects the other tau isoforms, and given their differences in primary structure, it is possible that phosphorylation may have differential impacts on their function. We have therefore characterized the polymerization and microtubule binding properties for each isoform, and the effect that phosphorylation by GSK-3β has on these properties.

We have found that there are fundamental differences in the functions of 3R and 4R isoforms. These differences are largely determined by the presence or absence of exon 10, with some contributions from exons 2 and 3. Phosphorylation by GSK-3β modulates the function of tau, but only mildly. The data suggest that phosphorylation alters all isoforms to approximately the same degree. These results suggest that the inherent properties of the isoforms are primarily responsible for their variable involvement in disease pathology, rather than the differential effects brought about by GSK-3β phosphorylation.

## Results

### Tau isoforms

**Figure 1:**
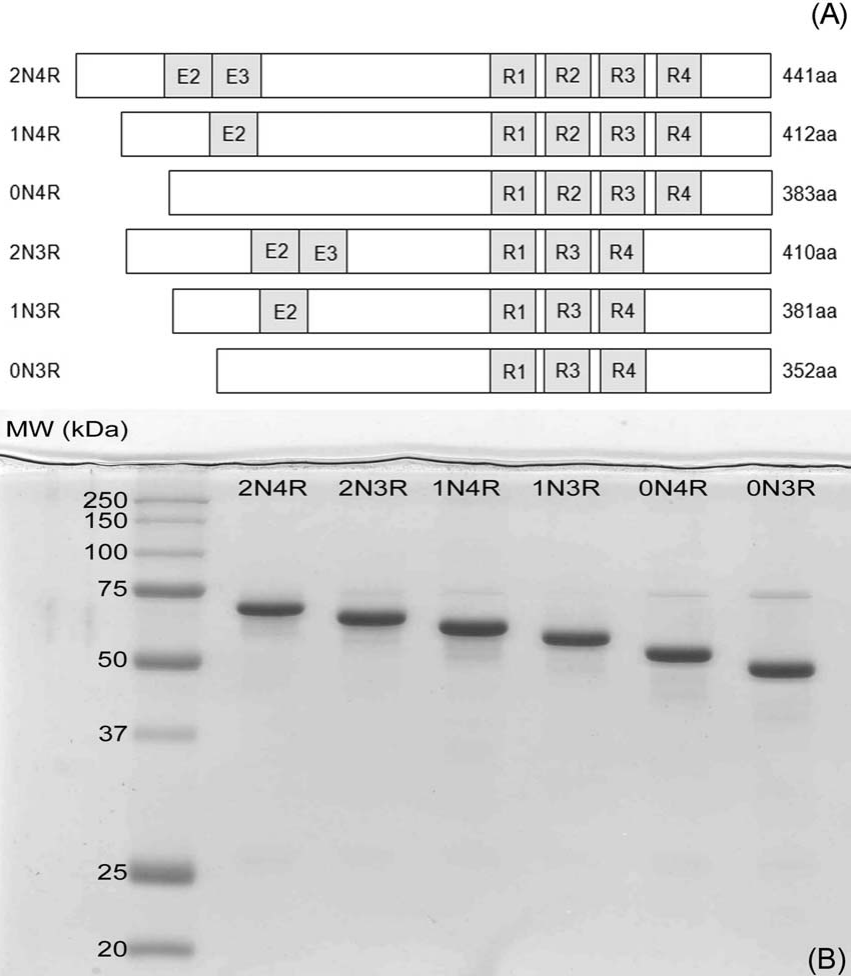
**Differences between tau isoforms**. (A) The six tau isoforms differ by the presence or absence of N-terminal exons 2 and 3 and the presence or absence of the C-terminal exon 10, which encodes microtubule binding repeat 2. Labels on the left indicate the isoform name, while labels on the right indicate the number of amino acids present in each corresponding isoform. (B) Each lane is labeled with the name of the isoform analyzed. Each isoform migrates a distinct distance on a Coomassie stained 10% SDS-polyacrylamide gel.

The tau transcript is alternatively spliced in humans to form six isoforms. These isoforms (0N3R, 0N4R, 1N3R, 1N4R, 2N3R, 2N4R) are classified by the presence or absence of N-terminal exons 2, 3, and C-terminal exon 10 (containing microtubule binding repeat 2) (Figure 1a). We expressed and purified each recombinant isoform from *E. coli *(Figure 1b). The isoform identity was confirmed by molecular weight determination on coomassie stained-sodium dodecyl sulfate polyacrylamide gel electrophoresis (SDS-PAGE) as compared to expected values in the literature [9,20].

### Isoform difference in arachidonic acid induced polymerization

**Figure 2:**
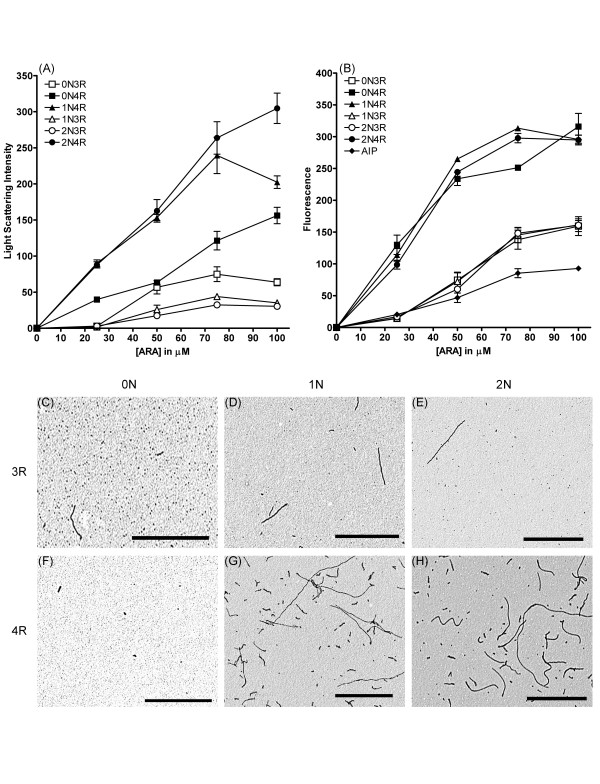
**ARA induction of tau isoform polymerization**. 2 μM tau isoforms were incubated with various ARA concentrations (0 μM to 100 μM) for 18 hrs. Polymerization was measured by (A) LLS or (B) ThS fluorescence. Symbols correspond as follows: (open square) 0N3R, (closed square) 0N4R, (open triangle) 1N3R, (closed triangle) 1N4R, (open circle) 2N3R, (closed circle) 2N4R, and (closed diamond) assembly incompetent protein (AIP (I277, 308P)). Data is in arbitrary units and represents an average of 3 trials ± SEM. After 18 hours, ARA induced polymerization reactions were visualized by TEM at 20,000x magnification. Images are as follows: (C) 0N3R, (D) 1N3R, (E) 2N3R, (F) 0N4R, (G) 1N4R, and (H) 2N4R. Each isoform image is representative of polymerized material at 75 μM ARA. Scale bars represent 1 μm.

Because the primary sequences of the tau isoforms are different, the various combinations of exons could influence their interactions with inducer molecules or cause conformational changes affecting tau-tau interactions during the polymerization process. The polymerization characteristics of each isoform was determined by incubating 2 μM protein with a range of ARA concentrations (0–150 μM) and monitoring the extent of polymerization with right angle laser light scattering (LLS, Figure 2a) and thioflavine S fluorescence (ThS, Figure 2b). We determined that above 100 μM ARA, the results were highly variable and not easily reproducible (data not shown). This is consistent with previously published results showing high variability at the inhibitory ratio of inducer to 2N4R tau [7]. The isoforms followed the same general trend of increasing amounts of polymerization with increasing inducer concentrations. 4R tau isoforms (containing exon 10) polymerized to a greater extent than 3R isoforms (Figure 2a & 2b), indicating that the additional microtubule binding repeat enhances tau polymerization.

The resulting filaments from the polymerization reactions above were viewed by transmission electron microscopy (TEM, Figure 2c–h). At equivalent ARA concentrations (75 μM), the greatest amount of filament formation was observed with the 1N4R and 2N4R isoforms (Figure 2g,h). Fewer filaments were observed for 1N3R and 2N3R (Figure 2d,e). Isoforms lacking both N-terminal exons (0N3R and 0N4R, Figure 2c, f) tended to form very small filaments (< 50 nm), as has been described previously [20], but large aggregates of longer filaments were also observed, albeit infrequently (Additional file 1). We, and others, have found that while aggregates of tau polymer can be detected by LLS and ThS fluorescence, these aggregates may not be uniformly distributed on a TEM grid, resulting in the user not being able to see these aggregates unless they scan the entire grid.

### Isoform differences in heparin induced polymerization

**Figure 3:**
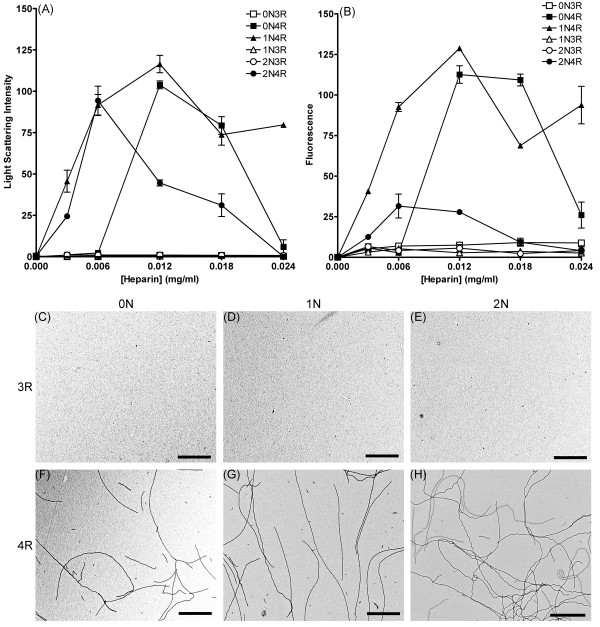
**Heparin induction of tau isoform polymerization**. 2 μM tau isoforms were mixed with various heparin concentrations (0 mg/ml to 0.024 mg/ml) and incubated 18 hrs. Polymerization was measured by (A) LLS or (B) ThS fluorescence. Symbols correspond as follows: (open square) 0N3R, (closed square) 0N4R, (open triangle) 1N3R, (closed triangle) 1N4R, (open circle) 2N3R, (closed circle) 2N4R. Data is in arbitrary units and represents an average of 3 trials ± SEM except for 2N4R with 0.006 and 0.018 mg/ml heparin, 1N4R with 0.024 mg/ml heparin, and 0N4R with 0.018 mg/ml heparin which were 2 trials. After 18 hours, heparin induced polymerization reactions were visualized by TEM at 20,000× magnification. Images are as follows: (C) 0N3R, (D) 1N3R, (E) 2N3R, (F) 0N4R, (G) 1N4R, and (H) 2N4R. Each isoform image is representative of polymerized material at 0.006 mg/ml heparin. Scale bars represent 1 μm.

Because it is known that some isoforms do not polymerize equally in the presence of heparin [21], the polymerization characteristics for all six isoforms were determined under conditions of low ionic strength [22,23]. The isoforms (2 μM) were incubated in the presence of varying concentrations (0–0.024 mg/ml) of heparin and polymerization was measured by LLS (Figure 3a) and ThS fluorescence (Figure 3b). Polymerization was detected by both methods for the 4R isoforms, and the dose-response curve was biphasic, indicating an optimal inducer concentration for polymerization. 2N4R required a lower concentration of heparin for optimal polymerization than 0N4R and 1N4R. Under these heparin induction conditions, the 3R isoforms failed to polymerize to detectable levels above background (Figure 3a and 3b). 4R isoforms (Figure 3f–h) were induced to form long filaments, but 3R isoforms (Figure 3c–e) did not polymerize into any observable filaments by TEM.

### Tau isoforms are phosphorylated by GSK-3β

All six tau isoforms are abnormally hyperphosphorylated in neurofibrillary tangles found in AD pathology. However the functional consequences of this phosphorylation are unknown. Previous studies have found that tau phosphorylation, in general, reduces its binding to microtubules [24]. Likewise, ARA induced filaments of GSK-3β phosphorylated 2N4R tau coalesce into "neurofibrillary tangle-like" structures [13,19]. To determine whether GSK-3β exerted similar effects on other tau isoforms, we examined the effects of GSK-3β phosphorylation by measuring the extent of phosphorylation, microtubule binding and ARA induced polymerization.

**Figure 4:**
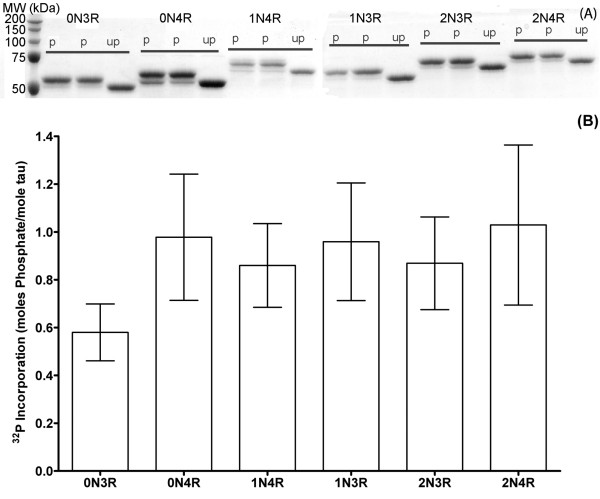
**GSK-3β phosphorylation of tau isoforms visualized by changes in gel mobility and phosphate incorporation**. (A) The migration of GSK-3β phosphorylated tau isoforms (p) was compared to unphosphorylated isoforms (up) by Coomassie-stained SDS-PAGE. The isoforms are labeled above their respective lanes. 2N4R was analyzed on a separate gel and then aligned by its relative migration to molecular weight standards. (B) γ-^32^P ATP was added to the phosphorylation reaction overnight and moles of phosphate per mole of tau was calculated by averaging 4 trials ± SEM. Each isoform in (B) is individually labeled underneath its respective value.

GSK-3β phosphorylation of 2N4R tau results in an upward shift in mobility on SDS-PAGE gels [19]. The effects of GSK-3β phosphorylation on the electrophoretic mobility of the other isoforms was examined by visualizing phosphorylation reactions of each isoform by SDS-PAGE (Figure 4a). All phosphorylated isoforms had an upward shift in electrophoretic mobility compared to its non-phosphorylated standard (Figure 4a). The quantity of the shifted tau band was unequal for the different isoforms, despite equivalent conditions for phosphorylation. One potential explanation for the differences in quantity could be that the degree of phosphate incorporation is not equal for all isoforms. Therefore, the levels of phosphorylation by GSK-3β were determined directly for each isoform by using γ-^32^P ATP. An average of approximately 1 mole phosphate was incorporated per mole of tau for each isoform (Figure 4b). Only 0N3R showed lower phosphate incorporation, but due to the variability in the reactions, this difference did not reach statistically significant levels. Therefore, the difference in electrophoretic mobility between phosphorylated and non-phosphorylated tau, is not likely due to significant changes in phosphate incorporation, but rather could be due to similar levels of phosphorylation resulting in different levels of SDS-resistant conformational changes in the isoforms. Alternatively, the difference in the quantity could be a result of experimental variability of phosphorylation by GSK-3β.

### Microtubule binding of tau isoforms changes with phosphorylation by GSK-3β

**Figure 5:**
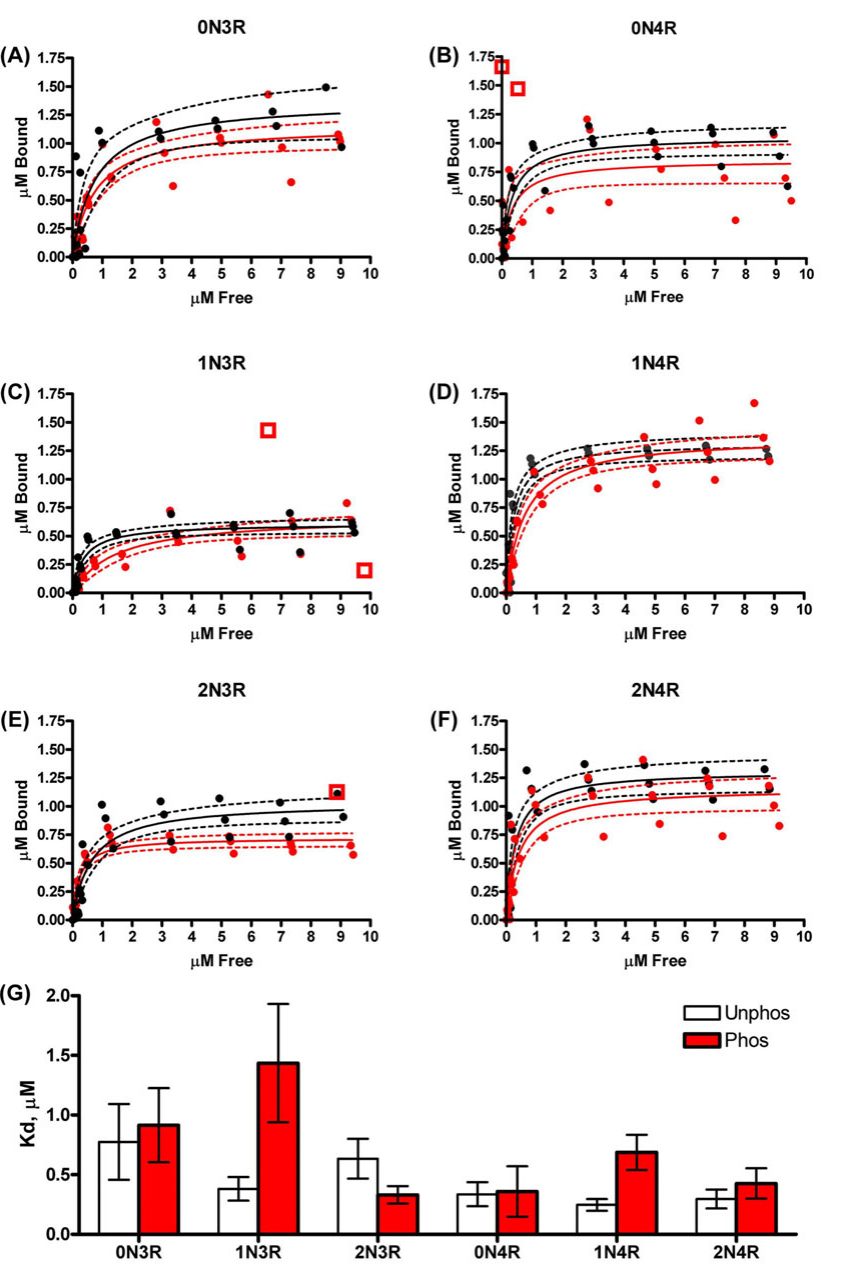
**Changes in microtubule binding of tau isoforms when phosphorylated with GSK-3β**. Microtubule binding reactions were performed at varying concentrations of tau (0 μM to 10 μM) and bound vs. free tau (in μM) for both unphosphorylated (black circle) and phosphorylated (red circle) was plotted (A-F). Three separate trials are plotted together to demonstrate the variability in the reactions. The data were fit to a nonlinear single site binding equation for unphosphorylated (black solid line) and phosphorylated (red solid line) tau isoforms (A-F). The 95% confidence intervals of the fits are drawn as dashed lines. Outliers existed for phosphorylated samples, are shown as red open boxes, and were excluded from calculations. (G) The dissociation constant (K_d_) (in μM) was determined for unphosphorylated (white bars) and phosphorylated (red bars) isoforms by K_d _= the concentration of tau required to reach 1/2 B_max_. Data represents the average of 3 trials ± SEM.

Tau is a microtubule-associated protein that, when phosphorylated at specific sites in the microtubule binding regions, such as at site S262 by p110^MARK ^(microtubule affinity regulating kinase), promotes strong disassociation (~10×) from microtubules [25], while phosphorylation outside of the microtubule binding regions (such as by GSK-3β in the proline rich region) has weaker effects on microtubule binding [26]. We therefore determined the effects of phosphorylation by GSK-3β on the microtubule binding affinities of each isoform, using unphosphorylated protein as a control (Figure 5a–f). Unphosphorylated 4R isoforms bound more tightly than the unphosphorylated 3R isoforms (Figure 5g), which is consistent with previously published results [27]. Upon phosphorylation, the largest decrease in microtubule affinities occurred in 1N isoforms (~3×) (Figure 5g), indicating GSK-3β phosphorylates 1N isoforms at sites that mildly influence microtubule binding. The average K_d _of phosphorylated 0N3R, 0N4R, and 2N4R isoforms was higher than the corresponding unphosphorylated K_d_, however, these changes did not reach statistically significant levels (Figure 5g). 2N3R showed an increase in affinity for microtubules upon GSK-3β phosphorylation. These data indicate that phosphorylation by GSK-3β has mild effects on the microtubule binding of tau isoforms, which is consistent with phosphorylation at the proline rich region or other regions only mildly involved with microtubule binding.

### Phosphorylation of tau isoforms changes filament polymerization levels

**Figure 6:**
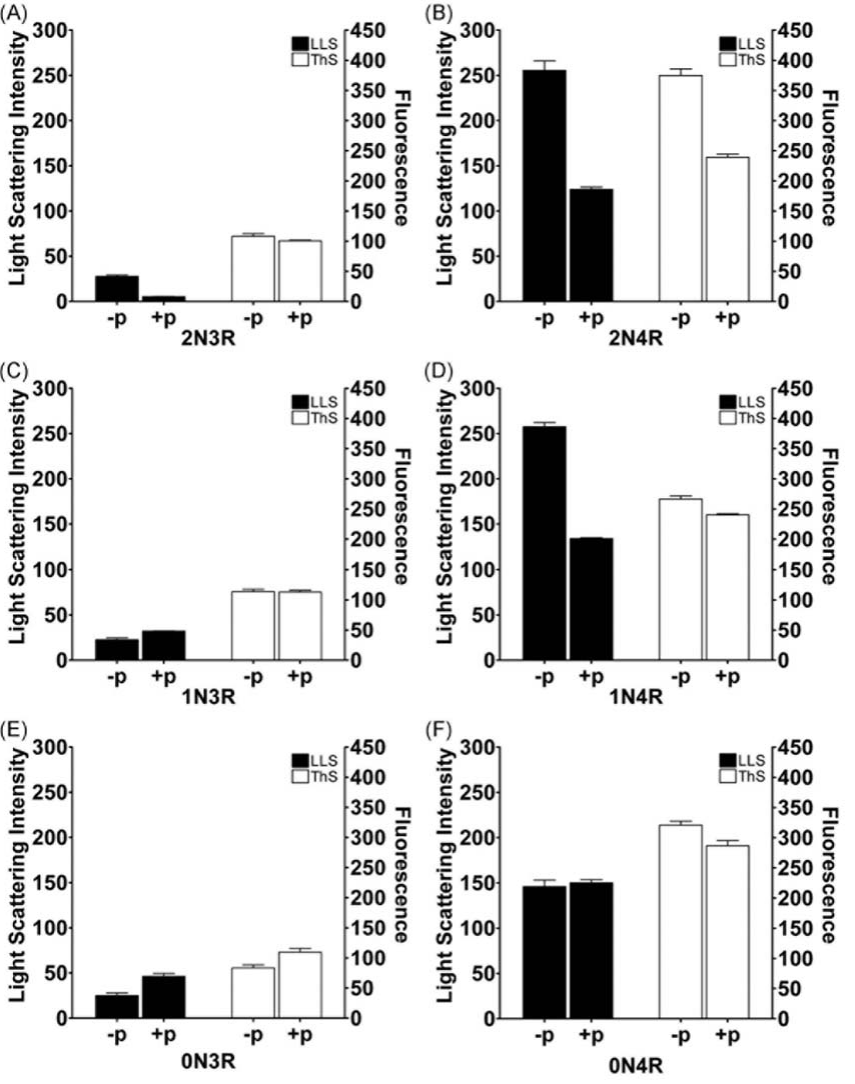
**Effect of phosphorylation on tau isoform polymerization**. Each isoform (A) 2N3R, (B) 2N4R, (C) 1N3R, (D) 1N4R, (E) 0N3R, and (F) 0N4R was polymerized with 75 μM ARA after phosphorylation by GSK-3β (+p) or without phosphorylation (-p). Reactions were measured by LLS (black bars and left y-axis) and ThS fluorescence (open bars and right y-axis). All LLS data is background subtracted with AIP for -p and AIPp for +p values respectively. Data is an average of 3 trials ± SEM.

To determine how phosphorylation affects polymerization with ARA, all six isoforms were phosphorylated prior to the induction of polymerization. Heparin was not used because it failed to induce polymerization in non-phosphorylated 3R isoforms (see Figure 3). The extent of polymerization at 2 μM tau and 75 μM ARA was measured by LLS and ThS fluorescence (Figure 6). Non-phosphorylated and phosphorylated assembly incompetent tau (AIP, I277/308P) was used to correct for background scattering as previously described [28]. 2N3R (Figure 6a) and 1N3R (Figure 6c) had relatively unchanged ThS values, but 2N3R had decreased LLS upon phosphorylation, while 1N3R had increased LLS. The phosphorylated 2N4R and 1N4R isoforms polymerized less than their unphosphorylated counterparts as measured by both LLS and ThS. Phosphorylated 0N3R had increases in both LLS and ThS, while 0N4R had no change in LLS, and decreased ThS. The data indicate that phosphorylation does not influence the polymerization of isoforms equally, but these changes are mild in comparison to differences between unphosphorylated isoforms.

### Phosphorylation of tau isoforms changes filament morphology

**Figure 7:**
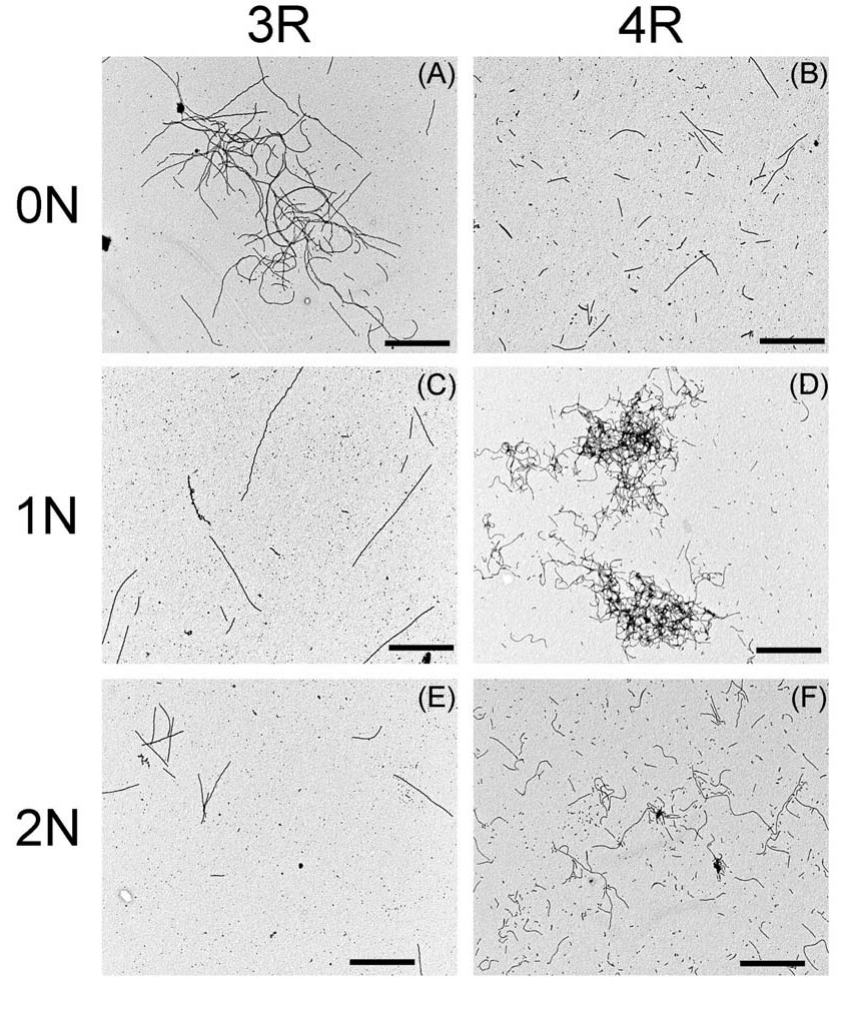
**TEM analysis of ARA induction of GSK-3β phosphorylated tau isoforms**. Images are as follows: (A) 0N3R, (B) 0N4R, (C) 1N3R, (D) 1N4R, (E) 2N3R, and (F) 2N4R. Images were recorded at a magnification of 20,000× (A-F) and are representative of polymerized material from 75 μM ARA induced-reactions presented in Figure 6. Scale bars represent 1 μm.

Because LLS readings can be influenced by the size and length of the particles [29] and it is not clear to which molecular species ThS binds [30-32], samples of phosphorylated tau isoform filaments were viewed by TEM to determine whether phosphorylation induces significant changes in filament morphology, as has been observed with 2N4R tau [13,19]. Representative micrographs of phosphorylated samples polymerized in the presence of 75 μM ARA indicate distinct morphological changes from unphosphorylated tau isoforms (Compare Figure 7 and Figure 2). Phosphorylated 0N3R and 0N4R (Figure 7a, b) isoforms had increases in the relative length and number of filaments formed as compared to non-phosphorylated (Figure 2c, f). Filaments formed from phosphorylated 1N3R and 1N4R (Figure 7c, d) isoforms also appeared to be relatively more numerous, and longer than non-phosphorylated (Figure 2d, g), although these changes were less dramatic than for the 0N isoforms. Phosphorylated 2N3R and 2N4R (Figure 7e, f) appeared to be relatively unchanged in the length and number of filaments (Figure 3e, h). Filaments from phosphorylated 0N3R, 1N4R, and 2N4R, had an increased tendency to cluster in close association to form "tangle-like" structures, similar to those seen previously with phosphorylated 2N4R induced with lower (25 μM) ARA concentrations [13,19]. It is apparent that isoform and inducer concentration, as well as phosphorylation, affect filament interactions promoting "tangle-like" structures.

## Discussion

Many studies aimed at understanding the normal and abnormal functions of the microtubule-associated protein tau have focused on only one or two of its isoforms. However, all six isoforms of tau differ in their primary structure and therefore can potentially differ in their functions. In fact it has been shown that significant differences exist between the microtubule binding and microtubule stabilizing capacities of isoforms with three microtubule binding repeats and those with four microtubule binding repeats [27,33]. It has also been demonstrated that there are substantial differences between the self-polymerization characteristics of three repeat isoforms and four repeat isoforms [20,21] and between isoforms with different numbers of N-terminal exons [20]. We have recently demonstrated that the phosphorylation of the 2N4R isoform of tau by GSK-3β causes arachidonic acid induced filaments to coalesce into larger structures similar to those purified from Alzheimer's disease [13,19]. Due to the potential differences between the characteristic functions of tau isoforms, we investigated the effects of GSK-3β phosphorylation on all six isoforms of tau.

As a necessary first step for this study, we established baseline parameters for the self-polymerization of non-phosphorylated tau isoforms. This step was necessary because it is clear the type of inducer used and the conditions for polymerization have a large influence on the degree of polymerization of tau isoforms [7,21-23]. Using arachidonic acid as an inducer for polymerization, we found several significant differences between the isoforms. First, four repeat isoforms polymerized to a greater extent than three repeat isoforms. Secondly, removal of the two N-terminal exons (exons 2 and 3) increased the concentration of inducer required for maximal polymerization. Lastly, the presence of exon 2 is sufficient to reduce the levels of inducer required for maximal polymerization because the addition of exon 3 to exon 2 containing isoforms does not further reduce inducer concentrations needed for maximal polymerization.

Similar results were obtained for the heparin induction of tau isoform polymerization. Four repeat isoforms polymerized in the presence of heparin while the amount of three repeat isoform polymerization was greatly reduced to undetectable levels. The N-terminal exons also influenced the concentrations of inducer required to reach maximal polymerization. The 2N4R isoform required less heparin inducer for maximal polymerization than 1N4R and 0N4R. The observation that three repeat isoforms essentially failed to polymerize in the presence of heparin was somewhat surprising because it has been shown that the heparin induced polymerization of 0N3R tau is actually enhanced as compared to 2N4R tau [21]. It should be noted that the aforementioned heparin polymerization studies were incubated at higher protein and inducer concentrations in solutions with greater ionic strength and for longer time periods than the current study. Our conditions for polymerization are based on previously published conditions of low ionic strength, protein concentration and inducer concentration that result in efficient tau polymerization reaching apparent steady state in 24–48 hours [22,23]. These results indicate that the local environment has a large impact on how individual tau isoforms self-polymerize, which could have an impact on isoform specific regulation of tau in the cell.

Our results and those of others strongly suggest that most isoforms of tau have distinct polymerization characteristics. The local environment is a major determinant of the amount of polymerization observed, and the isoforms do not respond equally to changes in environment. GSK-3β has been shown to phosphorylate tau in transgenic mice and cause neurodegeneration [34], which can be abolished with lithium treatment to inhibit GSK-3β [15]. Based on these observations, we examined GSK-3β phosphorylation of tau isoforms.

The levels of GSK-3β phosphorylation of the isoforms were similar at an average of approximately 1 mole of phosphate per mole of tau, although there was a large degree of variability in the amount of phosphate incorporation in repeated experiments. On average, the 0N3R isoform was phosphorylated to a lesser degree than the other isoforms, but not at statistically significant levels. Based on previous results, it is likely that phosphorylation occurs at more than one site. 2N4R tau phosphorylated by GSK-3β in vitro resulted in approximately 2–4 moles of phosphate per mole of tau distributed among eleven sites [12,13]. Phosphorylation by GSK-3β resulted in upward shifts in gel mobility assays, indicating that phosphorylation generates an SDS-resistant conformational change in each of the isoforms. Based on these data, it would be difficult to conclude that GSK-3β preferentially phosphorylates any particular isoform in vitro.

Although the level of GSK-3β phosphorylation was approximately equal, there were differences in the effects on isoform functions. As expected, four repeat tau isoforms bound more tightly to microtubules than did three repeat isoforms. Phosphorylation by GSK-3β decreased the average K_d _of 0N3R, 0N4R and 2N4R, but not at statistically significant levels due to the variability in the data. The 2N3R isoform bound more tightly to microtubules upon phosphorylation, which is not unprecedented for the effects of phosphorylation on tau-microtubule interactions [35]. The largest differences were observed for the 1N isoforms. Both 1N3R and 1N4R had approximately 3-fold reductions in microtubule binding following GSK-3β phosphorylation. While phosphorylation with GSK-3β affects microtubule binding differently for each isoform, these effects are small as compared to kinases (such as p110^MARK ^at S262) which cause a complete loss in microtubule binding of tau [25].

The effect of GSK-3β phosphorylation on the ARA induction of tau polymerization also differed between isoforms. In general, phosphorylation decreased the amount of detectable polymerization by ThS fluorescence for the 4R isoforms and had little effect on the polymerization of 3R isoforms. It is difficult to assess the amount of polymerization detected by LLS since the phosphorylation process seemed to increase the tendency of several isoforms to cluster into larger structures. These larger structures can potentially result in aberrant scattering [28,29,36,37]. The largest changes observed due to phosphorylation occurred with filament morphology as viewed by TEM. When compared to the unphosphorylated state, each isoform tended to increase in length and some showed increased affinities between polymers. However, the physiological relevance of such changes in morphology is unknown.

GSK-3β phosphorylation influences each of the tau isoforms differently, although these changes appear to be small in comparison to the inherent differences in the non-phosphorylated proteins. For example, GSK-3β phosphorylation reduces the ARA induced polymerization of the 4R isoforms, but the phosphorylated versions still polymerize to a greater degree than the non-phosphorylated 3R isoforms. GSK-3β phosphorylation of tau isoforms does alter their microtubule binding in a variable manner, but these changes are small, with the greatest effect on binding being an approximate 3-fold reduction in binding.

## Conclusion

In conclusion, GSK-3β phosphorylation has different impacts on the functions of the six isoforms of tau. However, the effect of GSK-3β phosphorylation on tau function appears to be less significant than the pre-existing differences in isoform function based on their primary structure. These inherent differences in the isoforms seem to react differently to the local environment which appears to be the major determinant of the amount of polymerization. However, GSK-3β was the only kinase examined in these studies, and there are numerous other kinases that are known to be involved in phosphorylating tau in vivo. Since we are only looking at unprimed sites, it is plausible that priming with other kinases before GSK-3β phosphorylation may have more profound effects on isoform function. This work is therefore a vital first step in understanding the effects of phosphorylation and hyperphosphorylation on the functional biochemical properties of tau isoforms.

## Methods

### Chemicals and Reagents

Arachidonic acid was obtained from Cayman Chemicals (Ann Arbor, MI), IPTG from Calbiochem (EMD Biosciences, La Jolla, CA), SDS-PAGE protein marker from Invitrogen (Gaithersburg, MD), thioflavine S and GSK-3β from Sigma (St. Louis, MO), urea from Bio-Rad (Hercules, CA), and uranyl acetate and formvar carbon coated grids from Electron Microscopy Sciences (Hatfield, PA). Tubulin and tubulin buffer components were from Cytoskeleton, Inc (Denver, CO). Constructs for the histidine tagged tau isoforms (0N3R, 0N4R, 1N3R, 1N4R, 2N3R and 2N4R) were a kind gift of Lester Binder. The construct for histidine tagged assembly incompetent tau (AIP (I277/308P)) was a kind gift from Jeff Kuret. Isoforms were expressed in *E. coli *and purified by nickel affinity chromatography using Chelating Sepharose Fast Flow (GE Healthcare Bio-Sciences, Corp, Piscataway, NJ) and size exclusion chromatography as previously described for 2N4R tau [19]. Protein concentration was determined by a commercial BCA assay (Pierce Chemical, Rockford, IL).

### Tau polymerization with ARA and heparin

Arachidonic acid (ARA) induction: Individual tau isoforms at a final protein concentration of 2 μM were incubated in polymerization buffer containing 10 mM Hepes pH 7.64, 100 mM NaCl, 0.1 mM EDTA and 5 mM DTT in the presence of varying concentrations of ARA (0 μM–150 μM, concentration of ethanol carrier was kept constant at 3.75% for all reactions) for 18 hours at 23°C. Reaction volumes were 200 μL.

Heparin induction: Individual tau isoforms at a final protein concentration of 2 μM were incubated in polymerization buffer containing 30 mM Hepes pH 7.64, 20 mM NaCl and 5 mM DTT induction reactions (200 μL total) in the presence of varying concentrations of heparin (0 mg/ml–0.024 mg/ml) for 18 hours at 37°C. Data for one of the three trials for 2N4R with 0.006 and 0.018 mg/ml heparin, 1N4R with 0.024 mg/ml heparin, and 0N4R with 0.018 mg/ml heparin were found to be inconsistent (greater than 33% different) from the other data and were not included in the graphs.

### Right Angle Laser Light Scattering

Tau polymerization reactions were incubated for 18 hours, and assayed by right angle laser light scattering as described in [7]. Briefly, reactions were illuminated with a 5 mW solid state 475 nm laser (B & W Tek, Inc., Newark, DE) and the resulting images of scattered light perpendicular to the incoming light were captured with a digital camera (Sony XC-ST270), imported into Adobe Photoshop and using the histogram function of the program assigned a value [7].

### Thioflavine S Fluorescence

Polymerization reactions were incubated for 18 hours, and assayed by ThS fluorescence as described in [7]. Briefly, ThS was added to reactions at a final concentration of 20 μM and fluorescence was measured at λ_ex _of 440 nm and λ_em _of 520 nm [7].

### Transmission Electron Microscopy

Polymerization reactions were prepared for TEM by methods previously described [7]. Briefly, reactions were diluted 1:5, fixed with 2% glutaraldehyde, placed on formvar-carbon coated grids (Electron Microscopy Sciences Hatfield, PA), and stained with 2% uranyl acetate (Electron Microscopy Sciences Hatfield, PA) [7]. Prepared grids were viewed with a JEOL 1200 EXII electron microscope, and images were captured with the MegaViewII imaging system (Soft Imaging System, GmbH Münster, Germany).

### GSK-3β phosphorylation of tau isoforms

GSK-3β (Sigma Aldrich, St. Louis, MO) phosphorylation reactions (25 μL) of each tau isoform contained 16.66 μM tau, 0.018 U GSK-3β per pmol of tau in buffer containing 40 mM HEPES, pH 7.64, 5 mM EGTA, 3 mM MgCl_2_, and 2 mM ATP. One unit is defined as the amount of GSK-3 required to catalyze the transfer of 1 pmol phosphate to tau in 1 min at 30°C in a reaction volume of 25 μl. Reactions were incubated for 20 hours at 30°C [19]. The degree of phosphorylation was determined by examining the reaction products by 10% SDS-PAGE.

### ARA polymerization of GSK-3β phosphorylated tau isoforms

25 μL phosphorylation reactions of the individual isoforms (see above) were diluted into ARA containing polymerization reactions, as previously described in [19].

### γ-^32^P ATP Phosphate incorporation

Tau protein was phosphorylated as detailed in [19]. Briefly, tau was phosphorylated with 2 μM ATP plus 3.125 μCi [γ-^32^P] labeled ATP (Perkin-Elmer, Boston, MA). Samples were filtered and washed to remove unincorporated γ-^32^P, then counted in a liquid scintillation counter (Packard 1600TR) [19].

### Unphosphorylated Microtubule Binding

Microtubule binding assays were performed using the commercially available Microtubule Binding Protein Assay Kit (Cytoskeleton, Inc., Denver, CO) using the manufacturer's protocol. Varying concentrations of tau (0 μM–10 μM) were mixed with a constant concentration of paclitaxel stabilized microtubules at a final concentration of 1.6 μM tubulin dimers and 20 μM paclitaxel in a 50 μL reaction with the general tubulin buffer supplied with the kit (80 mM PIPES, pH 7.0, 2 mM MgCl_2_, 0.5 mM EGTA). Samples were incubated at room temperature for 30 minutes, and centrifuged at 23°C in 7 × 20 mm polycarbonate Beckman centrifuge tubes in a Beckman TLA 100 rotor at 100,000 × g for 5 minutes to sediment the microtubules and tau bound to microtubules. The pellets were resuspended in 1 × SDS sample buffer (0.0625 M Tris, 2% SDS, 10% glycerol, 5% β-mercaptoethanol) and tau bound to microtubules were separated from the pelleted tubulin by electrophoresis. The SDS-polyacrylamide gels were scanned with a Hewlett Packard Scanjet 7400 C and the density of the tau contained in the pellet (bound tau) was determined using Adobe Photoshop and a tau standard curve on the same SDS-polyacrylamide gel. Isoforms 0N3R, 0N4R, 1N3R, and tubulin have similar electrophoretic mobility. In order to distinguish tau from tubulin bands, the protein were transferred to PVDF by standard western blot techniques and probed with tau 46.1 primary antibody (gift from Lester I. Binder) and goat anti-mouse horseradish peroxidase conjugated secondary (Pierce). Amersham ECL Plus Western Blotting Detection System was used to detect the secondary antibody (GE Healthcare). The PVDF membrane was visualized using the Kodak Image Station 4000R (Eastman Kodak Co, Molecular Imaging Systems, Rochester, NY). Band intensities were determined by the histogram function of Adobe Photoshop. The amount of free tau left in solution was determined by subtracting the amount of tau in the bound fraction from the total amount of tau added to the reaction and normalized to the band intensity of tubulin. The concentrations of tau free in solution versus bound tau were plotted in GraphPad Prism 4 and fit to a noncooperative single-site binding equation: Free = (B_max _* Bound)/(k_d _+ Bound). Binding reactions were performed in triplicate for all tau isoforms.

### Phosphorylated microtubule binding

Individual tau isoform phosphorylation reactions (92 μL), containing 18.11 μM final concentration of tau, 0.018 U GSK-3β per pmol of tau in buffer containing 40 mM HEPES, pH 7.64, 5 mM EGTA, 3 mM MgCl_2_, and 2 mM ATP were incubated for 20 hours at 30°C. Microtubule binding reactions were carried out as described above and separated by SDS-polyacrylamide gel electrophoresis. 0N3R, 0N4R, and 1N3R were transferred to PVDF by standard western blotting techniques as described above, and PVDF blots were treated with shrimp alkaline phosphatase (Roche) before blocking, to remove the phosphate groups that interfere with tau 46.1 antibody binding. Images of gels and blots were collected and processed by methods described above, except that due to the large concentration of tubulin we were unable to normalize the data to the corresponding band. Due to variable data, 5 outliers were excluded from calculations based upon being more than twice as different as the corresponding trials. These points are 0N4Rp trial 3 1 μM and 2 μM, 1N3Rp trial 1 8 μM and trial 2 10 μM, and 2N3Rp trial 2 10 μM.

## Competing interests

The authors declare that they have no competing interests.

## Authors' contributions

KV participated in the design of the study, performed the experiments described and drafted the manuscript. TCG conceived of the study, participated in its design and coordination and helped to draft the manuscript. All authors read and approved the final manuscript.

## Supplementary Material

Additional file 1 **Polymerization of 0N3R and 0N4R isoforms with 75 μM ARA into larger aggregates is seen, but infrequent**. 0N3R (A, C, E) and 0N4R (B, D, F) isoforms exhibit filament elongation and clustering under non-phosphorylated conditions. Reactions from Figure 2 were visualized by TEM at 20,000× magnification. Each image is from a different trial, and was found to occur at about 1–3 times per grid. Scale bars represent 1 μm. Click here for file
