# Supplementary figures and images for: GSK-3β phosphorylation of functionally distinct tau isoforms has differential, but mild effects
Source: Mol Neurodegener. 2009 May 2;4:18. doi: 10.1186/1750-1326-4-18 (PMC2683827; doi:10.1186/1750-1326-4-18)

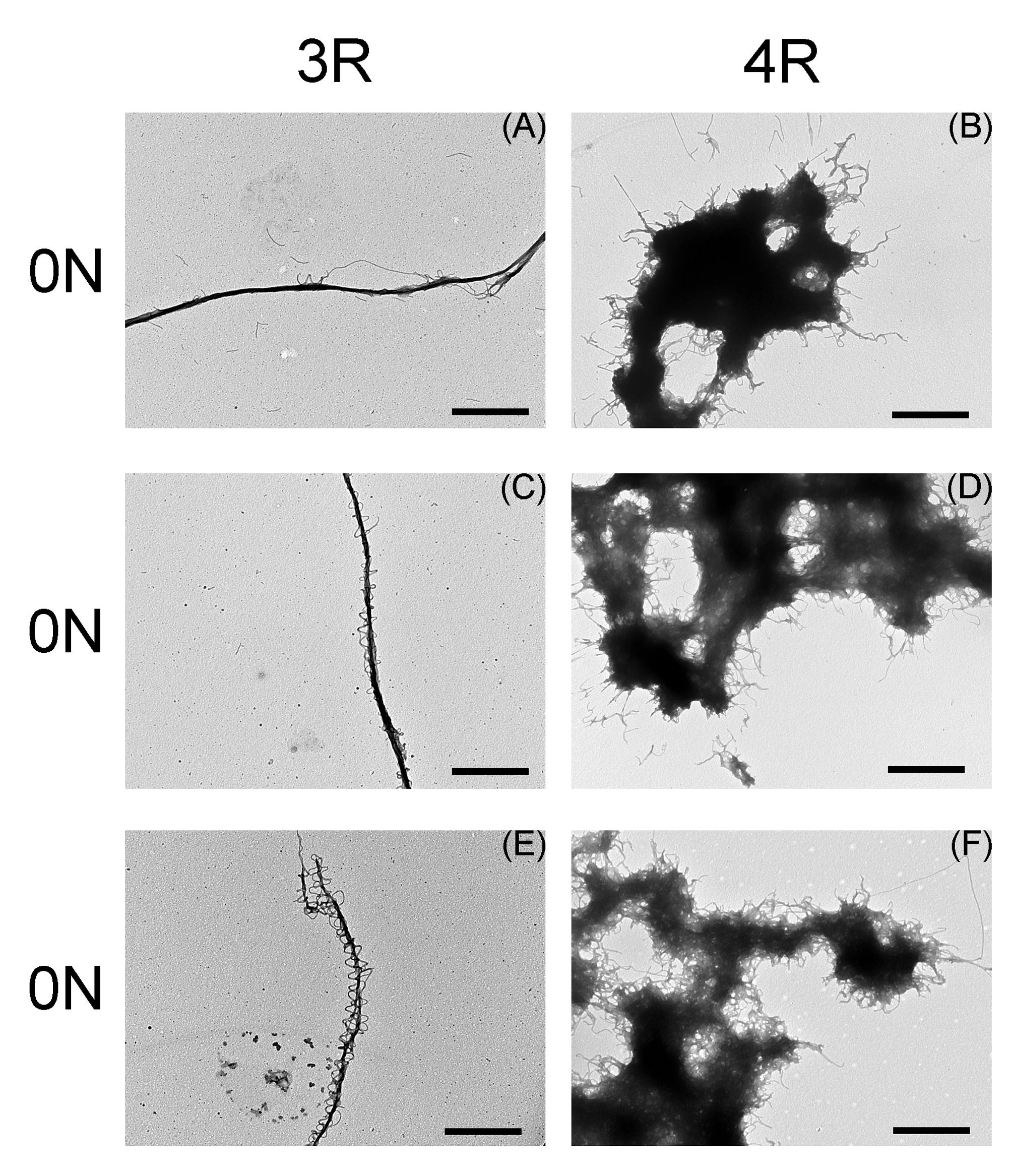

Supplement: Additional file 1 — Polymerization of 0N3R and 0N4R isoforms with 75 μM ARA into larger aggregates is seen, but infrequent. 0N3R (A, C, E) and 0N4R (B, D, F) isoforms exhibit filament elongation and clustering under non-phosphorylated conditions. Reactions from Figure 2 were visualized by TEM at 20,000× magnification. Each image is from a different trial, and was found to occur at about 1–3 times per grid. Scale bars represent 1 μm. [file 1750-1326-4-18-S1.tiff]
